# A Review of the Potential Use of Pinene and Linalool as Terpene-Based Medicines for Brain Health: Discovering Novel Therapeutics in the Flavours and Fragrances of Cannabis

**DOI:** 10.3389/fpsyt.2021.583211

**Published:** 2021-08-26

**Authors:** Katrina Weston-Green, Helen Clunas, Carlos Jimenez Naranjo

**Affiliations:** ^1^Neurohorizons Laboratory, Molecular Horizons and School of Medicine, Faculty of Science, Medicine and Health, University of Wollongong, Wollongong, NSW, Australia; ^2^Illawarra Health and Medical Research Institute, Wollongong, NSW, Australia; ^3^Australian Centre for Cannabinoid Clinical and Research Excellence (ACRE), New Lambton Heights, NSW, Australia

**Keywords:** cannabis, terpene, neuropharmacology and psychopharmacology, medicinal cannabis history, cannabinoid and terpene biosynthesis, pinene, linalool, cognition

## Abstract

“Medicinal cannabis” is defined as the use of cannabis-based products for the treatment of an illness. Investigations of cannabis compounds in psychiatric and neurological illnesses primarily focus on the major cannabinoids, cannabidiol (CBD) and Δ^9^-tetrahydrocannabinol (Δ^9^-THC), which are hypothesised to benefit multiple illnesses manifesting cognitive impairment, neurodegeneration and neuro-inflammation, as well as chronic pain, epilepsy and post-traumatic stress disorder, respectively. The cannabis plant contains >500 compounds, including terpenes responsible for the flavour and fragrance profiles of plants. Recently, research has begun providing evidence on the potential use of certain plant-derived terpenes in modern medicine, demonstrating anti-oxidant, anti-inflammatory, and neuroprotective effects of these compounds. This review examined the effects of two key terpenes, pinene and linalool, on parameters relevant to neurological and psychiatric disorders, highlighting gaps in the literature and recommendations for future research into terpene therapeutics. Overall, evidence is mostly limited to preclinical studies and well-designed clinical trials are lacking. Nevertheless, existing data suggests that pinene and linalool are relevant candidates for further investigation as novel medicines for illnesses, including stroke, ischemia, inflammatory and neuropathic pain (including migraine), cognitive impairment (relevant to Alzheimer's disease and ageing), insomnia, anxiety, and depression. Linalool and pinene influence multiple neurotransmitter, inflammatory and neurotrophic signals as well as behaviour, demonstrating psycho-activity (albeit non-intoxicating). Optimising the phytochemical profile of cannabis chemovars to yield therapeutic levels of beneficial terpenes and cannabinoids, such as linalool, pinene and CBD, could present a unique opportunity to discover novel medicines to treat psychiatric and neurological illnesses; however, further research is needed.

## Introduction: Medicinal Cannabis History, Entourage/Synergism

“Medicinal cannabis” is defined as the use of a cannabis-based product for the treatment of an illness (1). Patients can currently access cannabis products for medicinal purposes under the supervision of a medical specialist in many countries throughout the world; however, adequate data informing its prescription are still lacking. A number of factors contribute to the existing gaps in knowledge, such as a need for further well-designed clinical trials, reliable education, and training for prescribing clinicians, the stigma surrounding cannabis as a recreational drug, as well as policy issues, i.e., legalities governing patient access and possession of medical cannabis products, and laws governing legal production that can inadvertently affect safe and affordable supply. These issues can be attributed to the fact that cannabis remains an illegal narcotic drug of abuse due to its listing in the United Nations Single Convention on Narcotic Drugs Act (Schedule 1, 1961), although recently removed from Schedule 4 in acknowledgement of its medicinal value. In addition, biological factors, such as a continually developing understanding of the mammalian endocannabinoid system, coupled with the complex composition of the cannabis plant add further obstacles. Indeed, the complexity of the cannabis plant was observed by ancient civilizations earlier than 2000 BC, with documented reference to the word “MāFěn” [the Chinese word for cannabis, likely to be *Cannabis sativa* L. indigenous to Central Asia (2, 3)] described by emperors in Chinese Pharmacopoeia “Shen Nung Pen Ts'ao Ching” written in 100BCE that documented more than 2,000 years of Chinese agricultural and medicinal plant history (4). It was noted as a drug with the peculiar properties of both the “yin and yang”—possessing both the masculine and the feminine, or dark and light (4, 5). More than 4,000 years later, the complexity of cannabis remains a challenge, with a growing list of more than 500 chemical constituents identified in the plant, including cannabinoids and terpenes, as well as other compounds, such as flavonoids, vitamins, fatty acids, sterols, lignanamides, spiroindans, and alkaloids that may have health benefits (1, 6, 7).

The challenge for future research into the use of cannabis for human health benefits is confounded further by the suggested existence of an “entourage effect,” meaning that the combination of cannabis plant chemicals can act synergistically and potentially render whole plants extracts more effective than the individual products isolated from them. For example, in one study, a high CBD/low THC plant extract increased intracellular Ca^2+^ signalling in cultured hippocampal neurons compared to purified CBD (8), suggesting enhanced effects due to other plant compounds compared to CBD alone. A meta-analysis containing analyses of 670 patients across 11 studies revealed greater improvement in patients with treatment-resistant epilepsy using CBD-rich plant extract (71%) compared to CBD (37%) (*p* < 0.0001), with patients requiring a lower average dose (6.0 vs. 25.3 mg/kg/day in the whole plant extract group vs. CBD alone) and reporting less frequent adverse effects than those using purified CBD (9). Nevertheless, when the standard clinical threshold of a “50% reduction or more in the frequency of seizures” was applied, only 39% of the individuals were considered “responders,” and the difference between treatment with CBD-rich extracts (122/330, i.e., 37%) and purified CBD (94/223, i.e., 42%) was no longer significant (*p* = *0.52*) [corrected]. Based on recent *in-vitro* evidence in cell lines (10, 11), the popular notion that an “entourage” of cannabinoids and terpenes amplifies the effect of the major cannabinoids, THC and CBD, on the endogenous cannabinoid CB_1_ or CB_2_ receptor subtypes appears to be incorrect; however, studies in brain tissue are still needed to understand effects *in-vivo*. Whether synergism occurs via amplified effects on a single target, through effects of multiple different plant compounds on many targets, or by different phytochemicals activating endogenous interactions between the endocannabinoid system and other systems [e.g., dimerization of the cannabinoid CB_1_ receptor to dopaminergic D_2_ receptors (12)] is also unclear. Furthermore, although the plant itself is officially named *Cannabis sativa* L. (*C. sativa* L.) (after Swedish botanist Carl Linneaus), medicinal cannabis can appear as somewhat of a dynamic target as breeders continue to produce different strains/cultivars that can have vastly different effects on the body, likely due to differences in the relative levels of plant phytochemicals (i.e., different chemovars). On the other hand, the overall complexity of cannabis and its interactions in the body present unique opportunities for the discovery of novel personalised therapeutics, as certain cannabis strains may confer greater benefits for particular clinical indications.

Research to-date has mostly focused on the cannabidiol (CBD) and (–)-*trans*-Δ^9^-tetrahydrocannabinol (THC) content in plants, or formulations based on these major cannabinoids. Studies examining strains containing appreciable levels of THC reported some beneficial effects in treating illnesses such as (but not limited to) refractory paediatric epilepsy, anorexia, and wasting associated with chronic illness, muscle spasticity associated with multiple sclerosis, chronic pain, chemotherapy-induced nausea and vomiting, and post-traumatic stress disorder (13, 14). Medicinal cannabis is currently approved for most of the aforementioned indications in Australia, New Zealand, Canada, some states in USA, and a number of countries throughout Europe, Asia and South America. Conversely, use of certain chemovars of *C. sativa* L. as a whole plant product may exacerbate symptoms of mental health disorders in some people, particularly high THC, low CBD strains (15–17). It can also increase the likelihood of developing mood or psychiatric illnesses (18–20). On the other hand, we previously revealed a body of pre-clinical and clinical evidence suggesting that CBD induces a range of therapeutic benefits in psychiatric and neurological illnesses, including anxiolytic, anti-depressant, antipsychotic, neuroprotective, anti-inflammatory, anti-oxidant, and pro-cognitive effects [reviewed in Weston-Green (1) and Osborne et al. (21)]. The evidence included the ability of CBD to prevent cognitive harm and hippocampal volume loss in chronic cannabis users, improve cognition and pathological markers in models of Alzheimer's disease, improve psychiatric scores in patients with schizophrenia and Parkinson's disease, and confer neuroprotection and improve cognition in rodent models of hypoxia, ischemia, stroke, hepatic encephalopathy and sepsis [reviewed in Weston-Green (1) and Osborne et al. (21)]. Therefore, although evidence shows that cannabis can harm the brain under particular conditions, there is rapidly emerging research to show that certain components of this complex plant could lead to the discovery of novel treatments for serious illnesses of the brain that currently lack effective treatments. Terpenes are also a major component of cannabis; however, whether this group of compounds provide benefits for brain health is unclear.

## Methods

This review article examined studies investigating the effect of key terpenes, pinene and linalool, on parameters relevant to psychiatric and neurological disorders. A search of abstract, title and keywords was conducted across Pubmed and Web of Science and all relevant English articles (until July 2020) were reviewed. Key words included terpenes (pinene or linalool) and the following parameters: oxidation, inflammation, neuroprotection, pain, analgesia, depression, anxiety, cognition, learning, memory, sleep or insomnia, psychiatric, neurological. Wildcards were used to detect articles with differences in spelling e.g.,: oxidat*, depress*, psych*.

## Terpenes—Simply Flavours and Fragrances…(?)

Terpenes are the most abundant class of naturally occurring small molecules by mass on the planet (22). These compounds are not only present in plants, but have innumerable functional and structural roles in most life forms on Earth; including animals, fungi, marine organisms, insects, protozoa, and bacteria (23). For example, cholesterols are terpenes that are fundamental for cell signalling and lipid membrane structure, carotenoids are critical in plant photosynthesis, retinal is a terpene found in the eye that plays a role in vision, while quinone (co-enzyme Q) is an electron carrier necessary to drive cellular respiration (22).

### Terpene and Cannabinoid Nomenclature and Biosynthesis

**Figure 1:**
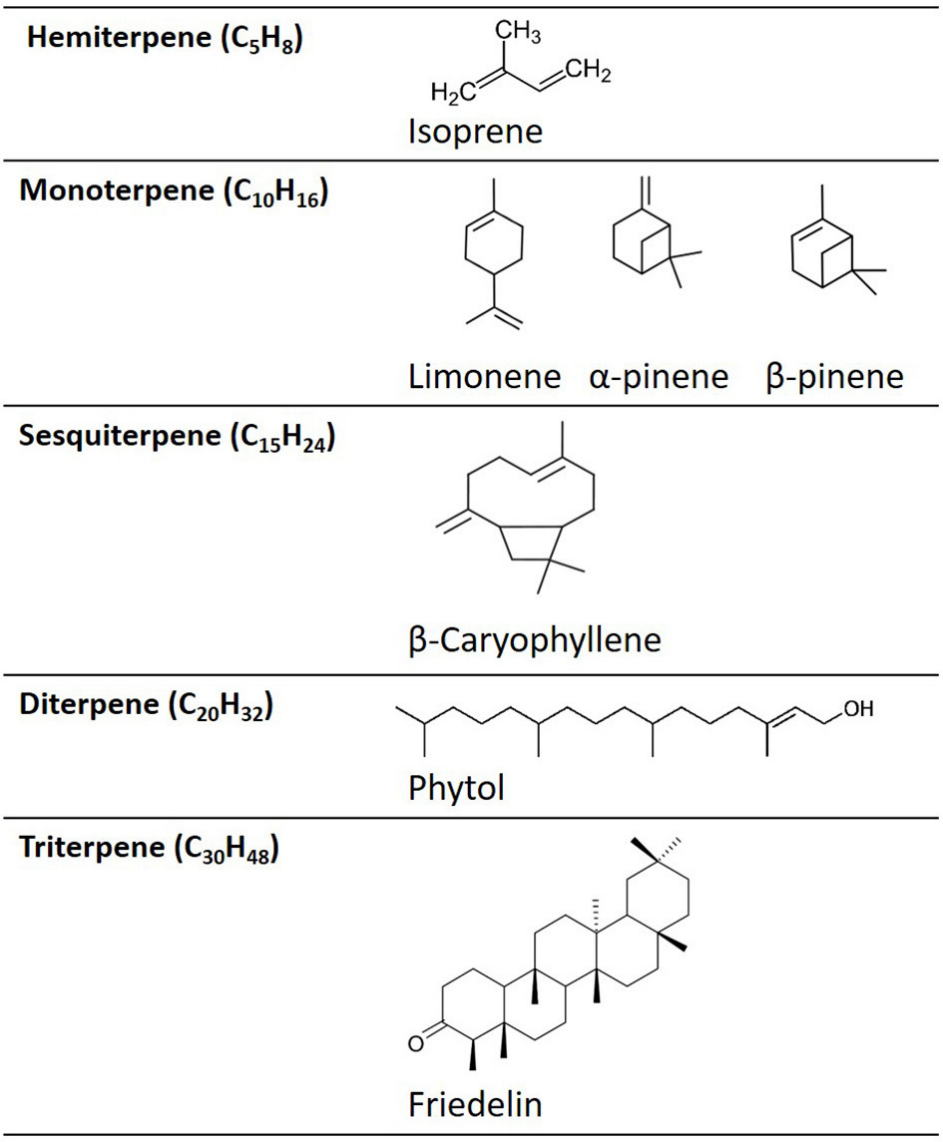
Examples of the chemical structures of different terpene categories.

Terpenes are comprised of two isoprene units (each C_5_H_8_) (Figure 1). In terpene nomenclature, the number of carbon atoms in the terpene backbone determines categorisation as a hemi-, mono-, sesqui-, di-, tri-, or tetra- terpene (24). For example, monoterpenes are hydrocarbons with the formula, C_10_H_16_, e.g., limonene, pinene, and linalool (Figures 1, **5**, **6**), while hemi-terpenes (*hemi* from Greek: half) contain a single isoprene unit, or half the number of carbons of a monoterpene (C_5_H_8_). Sesquiterpenes contain three isoprene units, with the formula C_15_H_24_ (*sesqui* from Latin *semi* + *que*: one and a half) e.g., caryophyllene, while di-, tri-, and tetra-terpenes contain 20, 30, and 40-carbon atoms, respectively (Figure 1). Terpenes can exist as cyclic or acyclic structures. Combining cyclic terpenes with other ring structures (e.g., cyclobutane to form caryophyllene) can result in the formation of two or more rings, referred to as bi-, tri-cyclic, etc. Furthermore, the addition of functional groups, such as oxygen, yields terpenoids (such as caryophyllene-oxide (BCP-oxide).

**Figure 2:**
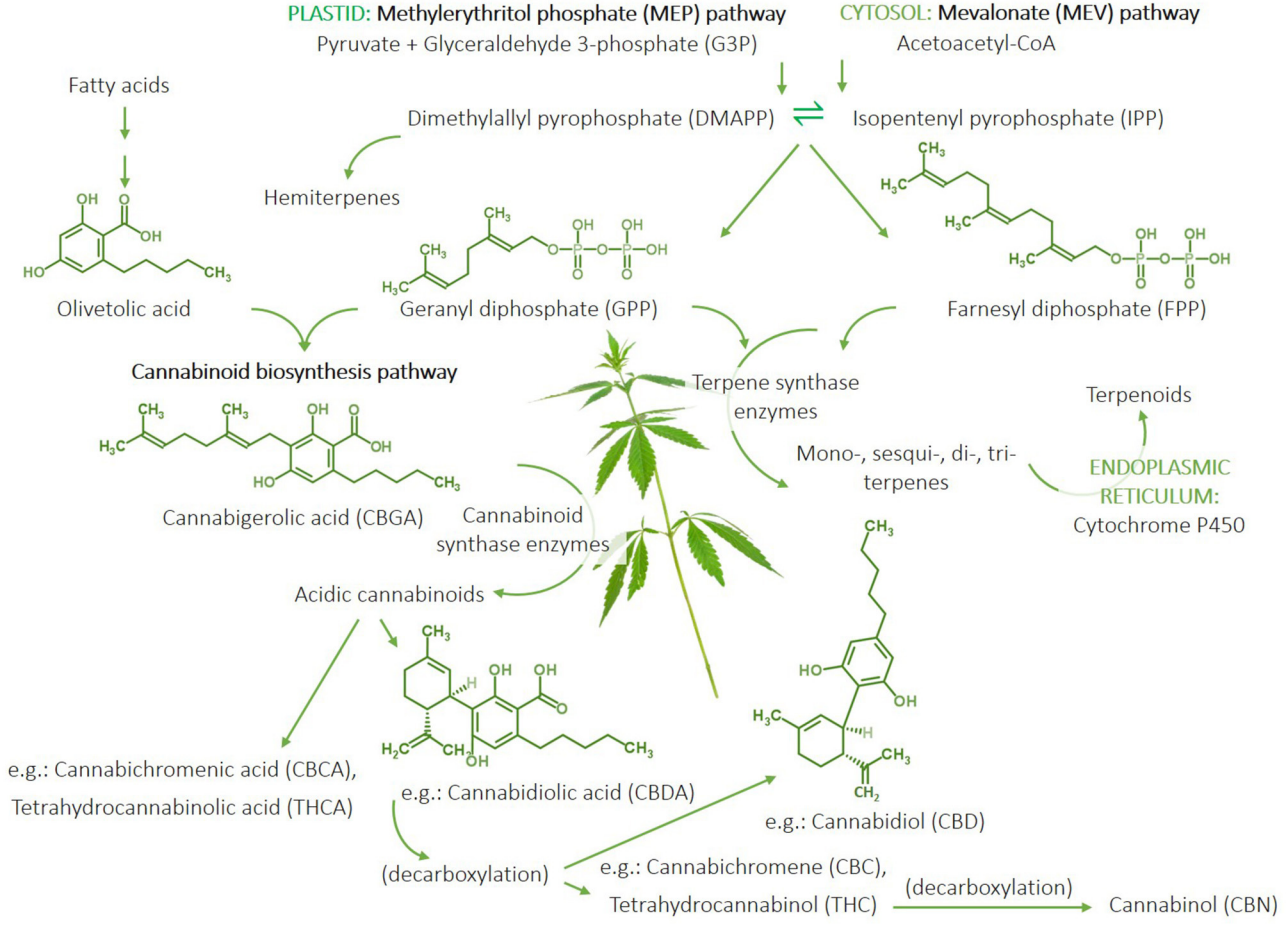
Summary of the terpene and cannabinoid biosynthetic pathways in cannabis plants. The methylerythritol phosphate (MEP) pathway occurs in the plastid and generates isoprenoid pre-cursors, isopentenyl pyrophosphate (IPP), and dimethylallyl pyrophosphate (DMAPP) from pyruvate and glyceraldehyde-3-phosphate. IPP and DMAPP can be used to produce hemiterpenes and geranyl diphosphate (GPP), which can lead to monoterpene and diterpene synthesis. GPP combined with olivetolic acid generated from fatty acids initiates the cannabinoid biosynthesis pathway starting with the production of cannabigerolic acid (CBGA) and subsequent acidic cannabinoids, including cannabidiolic aicd (CBDA), cannabichromenic acid (CBCA), and tetrahydrocannabinolic acid (THCA), catalysed by respective cannabinoid synthase enzymes produced by the plant. A percentage of the native acidic cannabinoids metabolise through decarboxylation to yield non-acidic by-products, e.g., cannabidiol (CBD), cannabichromene (CBC), and tetrahydrocannabinol (THC); the latter of which can be further metabolised to cannabinol (CBN). The cytosolic mevalonate (MEV) pathway also produces DMAPP and IPP from acetoacetate-CoA-derived precursors to generate farnesyl diphosphate (FPP), which can be used to generate sesqui- and triterpenes. Terpenes can be converted to terpenoids via the P450 enzyme in the endoplasmic reticulum. The production of mono-, sesqui-, di-, and triterpenes involves reactions catalysed by a multitude of terpene synthase enzymes expressed by the plant to yield an abundance of different terpene compounds. Therefore, the cannabinoid and terpene profile of a cannabis plant is largely determined by the expression of its synthase enzymes. “Cannabis Leaves Weed Png” by Clipart.info is licenced under CC BY 4.0.

Terpenes and cannabinoids are synthesised by plants through three major pathways (Figure 2). [1] The methylerythritol phosphate (MEP) pathway, which occurs in the plastid (i.e., the photosynthetic centre) of the plant cell, utilises pyruvate and glyceraldehyde-3-phosphate (G3P) to produce intermediaries that yield the isoprenoid precursors, dimethylallyl pyrophosphate (DMAPP), and isopentenyl pyrophosphate (IPP) (which can produce hemiterpenes), and subsequent geranyl diphosphate (GPP), the precursor to monoterpenes and separate intermediaries generating diterpenes and carotenoids. [2] GPP also combines with olivetolic acid generated from fatty acids to initiate the first step in the cannabinoid biosynthesis pathway that produces cannabigerolic acid (CBGA). CBGA is converted into the acidic forms of cannabinoids, e.g., cannabichromenic acid (CBCA), tetrahydrocannabinolic acid (THCA), cannabidiolic acid (CBDA), using the respective plant synthase enzymes (e.g., CBDA-synthase). Decarboxylation results in the conversion of the native acidic forms of the cannabinoids into the non-acidic metabolites, e.g., cannabichromene (CBC), CBD, THC; the latter of which can be further metabolised (decarboxylated) into cannabinol (CBN) (Figure 2). Therefore, the native acidic forms of cannabinoids are direct products of the plant, whereas the plants indirectly produce the non-acidic cannabinoids as metabolic by-products. [3] The mevalonate (MEV) pathway occurs in the cytosol of the plant cell and involves the conversion of acetoacetyl-CoA into the DMAPP and IPP intermediaries forming farnesyl diphosphate (FPP), which yields sesqui- and triterpenes. The reactions converting GPP and FPP into the various terpenes are catalysed by an array of terpene synthase enzymes. Furthermore, cytochrome P450 enzyme expressed in the endoplasmic reticulum catalyses the conversion of terpenes into terpenoids. These biosynthetic pathways and the metabolic products of their biosynthetic compounds, result in an enormous diversity of terpenes and cannabinoids, as well as their metabolites (28) (Figure 2).

The expression of terpene synthase genes can vary significantly between cannabis chemovars, for example Booth et al. (25) reported differences in terpene synthase gene profiles across eight cannabis strains (Lemon Skunk, CBD Skunk Haze, Blue Cheese, Afghan Kush, Chocolope, Blueberry, Vanilla Kush, and Jack Herer), and variations based on organ (leaf vs. flower) and phenological stage (maturation). Interestingly, the study reported 13 novel cannabis terpene synthase genes that had not previously been characterised, highlighting the ongoing development of scientific knowledge of terpenes in the cannabis plant. Variability in the genetic profile of plants and terpene synthases, combined with variations in the level of expression, and activity of resultant protein enzymes (e.g., due to varying environmental stimuli) are likely to underscore the variations in fragrance and flavour profiles of cannabis plants. In plants, the main function of terpenes is to shape the interactions between organisms, either promoting bilateral beneficial responses, such as attractants for pollinating insects, or triggering antagonistic reactions as toxins and repellents to protect against pathogens like mould, fungus, and bacteria (29, 30), or predation (31). Therefore, terpenes play a critical role in the normal functioning of plants. Interestingly, mounting evidence suggests that terpenes may be an important avenue for novel drug discovery, particularly medicines for neuropsychiatric and neurological illnesses that generally lack highly efficacious medications, highlighting the importance of understanding terpene profiles of cannabis plants that extends further than an appreciation of flavour and fragrance profiles.

### Terpenes as Medicines—Historical Overview

**Figure 3:**
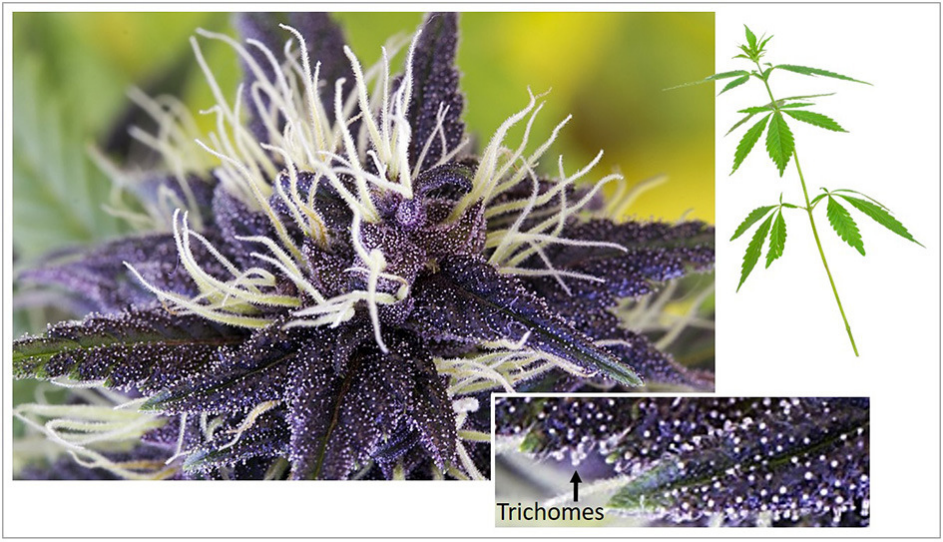
Female inflorescence of cannabidiol-rich Medi-Charlotte cannabis cultivar. Magnified inset shows glandular trichomes (white dots noted at arrow). GHM Genetic Development, Amsterdam, The Netherlands (2018).

Terpenes are secreted by most plant organs (from roots to stem, leaves, and flower), but are particularly rich in resins (32). The resin of cannabis is primarily produced in glandular structures called trichomes, which form abundantly in the female inflorescence (the flower, bract/calyx, stigma, and associated stem) and also in leaves and stem that are proximal to the flowering head (Figure 3). The cannabis plant mainly contains monoterpenes and sesquiterpenes (33, 34), although others, such as triterpenes, have been detected in hemp roots, fibres, and hemp seed oil (35).

The extraction of oils from leaves, flowers, fruit and roots of plants has been conducted by ancient civilizations since the beginning of human records [see (36) for review]. Therefore, terpenes have been used by humans in balms, oils and resins for various ailments for thousands of years. For example, oil of turpentine (sourced from *Pistacia terebinthus* L.) was commonly used by the Ancient Greeks; extractors of which were considered to hold reputable occupation (37), while use of other traditional plant based medicines have been recorded throughout Ancient India (Ayurveda), China and Rome, including frankincense, myrrh, and other balms that were recorded throughout Biblical times. Some well-known examples that continue mainstream popularity for use in various ailments include anti-bacterial tea tree oil (*Melaleuca alternifolia* (Maiden & Betche) Cheel) (38), *Ginkgo biloba* L. leaf for memory (39), anti-inflammatory curcumin from turmeric (*Curcuma longa* L.) (40) and Echinacea (*Echinacea purpurea* (L.) Moench) for treating the common cold (41). There are now over twenty-five thousand known terpenes and science is beginning to unravel the evidence both for and against their use in health, as well as their mechanisms of action.

**Figure 4:**
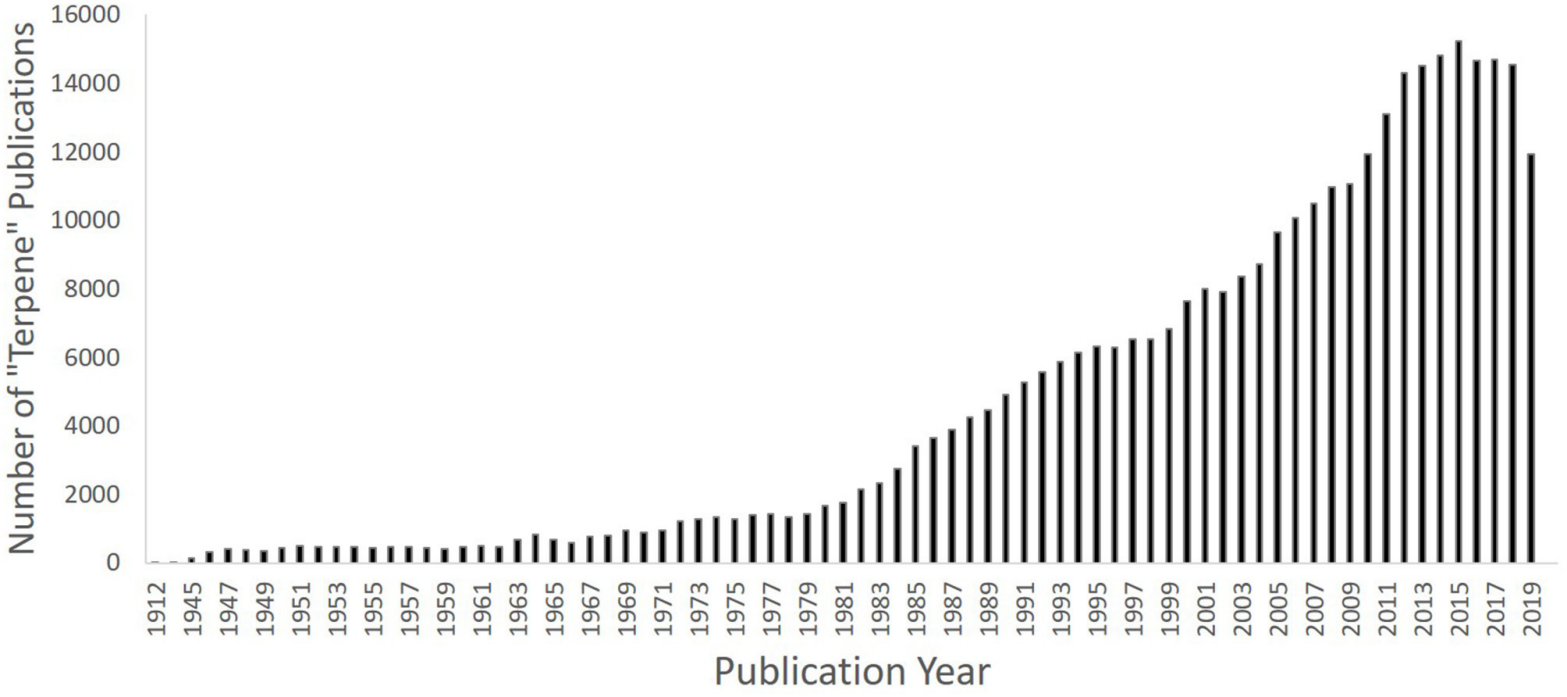
Graph depicting number of “terpene” publications since the first article in 1912, showing increase since the 1980s (past 30 years) until 2019 using PubMed as an example (PubMed [Internet]. Bethesda (MD): National Library of Medicine (US), National Center for Biotechnology Information; 2004–[cited 2020 July 13]. Available from: https://pubmed.ncbi.nlm.nih.gov.

The first terpene-related article documented in Pubmed was published in 1912 in the journal Science, by Professor G. B. Frankeforter on the relationship between resins and their constituent terpenes. Frankeforter (42) discussed “recent” landmark discoveries in the field of plant-based organic chemistry dating from the 1860s, and remarked that “even the resins, which chemists have until recently regarded as too complex to deserve serious attention, were studied in an industrial way and more than thirty different varieties prepared and used in the arts” (42). Indeed, it could be said that studying terpenes remains complex to-date, as the scientific community seeks to understand their pharmacological profiles (10, 11, 43), with an explosion of terpene research publications now evident, particularly over the past 30–40 years (Figure 4). These molecules continue to be used as safe additives in industry, for example as fragrances and flavours in food, textiles, and cosmetics; characteristics attributed to their low molecular weight and volatility. However, mounting *in-vitro* and *in-vivo* scientific evidence now shows that terpenes possess an array of medicinal benefits, particularly relevant to brain health. For example, extracts of maral root (Leuzea carthamoides Willd.), which contains several sesquiterpenes, improve learning and memory in rats (44), extracts from Cecropia species and valerian (*Valeriana officinalis* L.) elicit anxiolytic effects in mice (45, 46), and essential oils (e.g., bergamot, frankincense, ylang ylang, geranium, and rose) can reduce blood pressure, heart rate and glucocorticoid levels through effects on the hypothalamic-pituitary-adrenal axis [see review by Lizarraga-Valderrama (47)].

## Pinene

**Figure 5:**
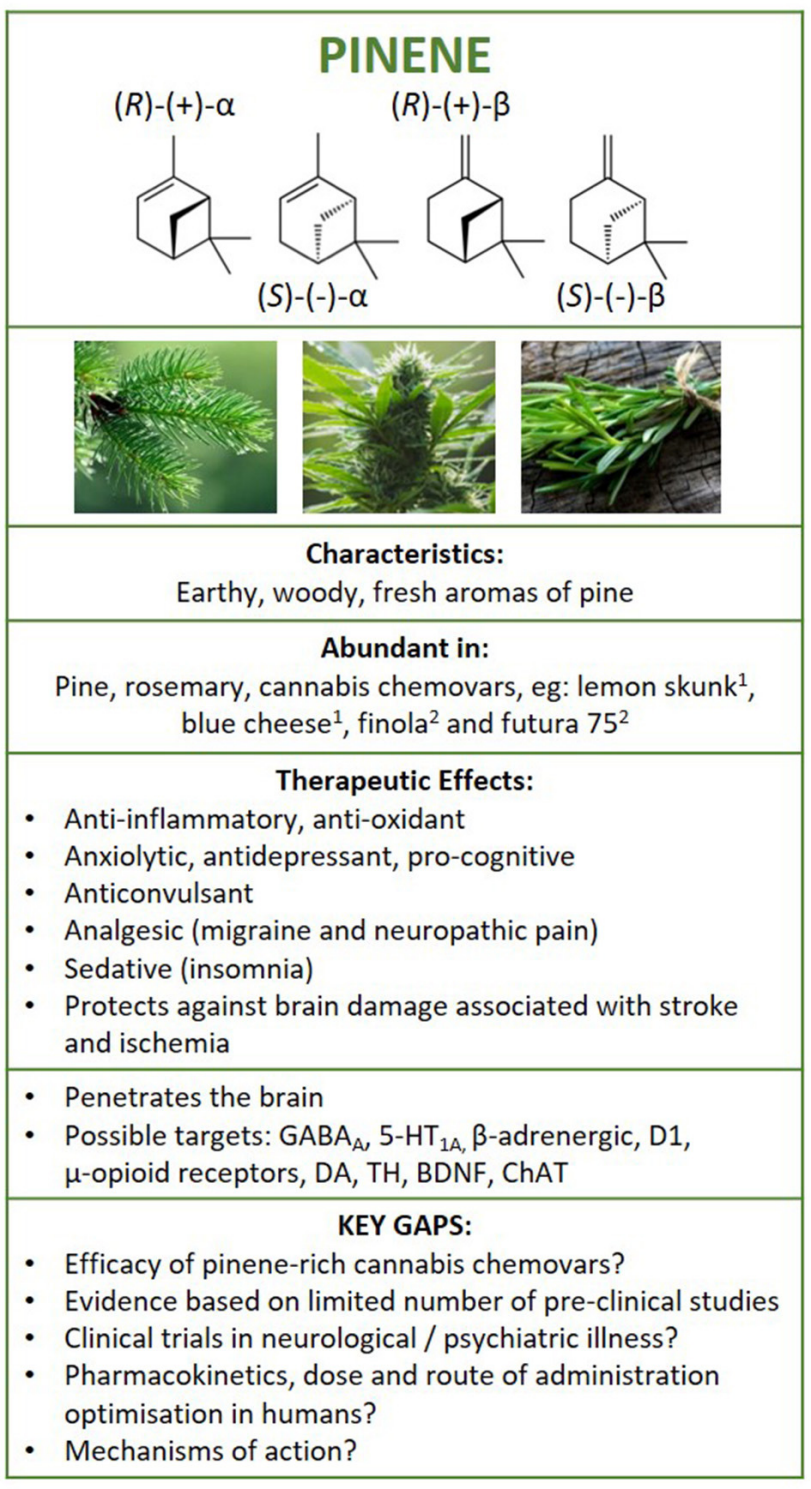
Structure of pinene isomers and enantiomers. Summary of findings following literature search on the therapeutic effects of pinene in neurological and psychiatric illness, and brain health. ^1^Booth et al. (25) and ^2^Pavlovic et al., (26). GABA_A_, γ-aminobutyric acid A receptor; 5HT1A, serotonin 5-HT1A receptor; D1, dopamine D1 receptor; DA, dopamine; BDNF, brain-derived neurotrophic factor; TH, tyrosine hydroxylase; ChAT, choline acetyltransferase.

Pinene is one of the most abundant terpenes in nature (48). It is a clear liquid associated with the earthy, woody, fresh aromas of pine, and resin found in many non-edible parts of plants (49). In nature, two structural isomers of this terpene exist, α-pinene and β-pinene (Figure 5) (50). α-pinene and β-pinene are the prominent terpenes in many *C. sativa* L. chemovars (26, 51–53). For example, one study reported average α-pinene and β-pinene concentrations of 15 and 21% across 17 hemp varieties (Antàl, Bielobrzerski, Carmagnola, Carmagnola CS, Dioica 88, Fedora 17, Ferimon, Finola, Futura 75, KC Virtus, KC Zuzan, Markant, Santhica 27, Santhica 70, Tiborszallasi, Tygra, and Zenith) (54). Another study reported high levels of α-pinene in Lemon Skunk and CBD Skunk Haze, while β-pinene was dominant in Purple Kush (25). These terpenes are also the major constituents of turpentine, and are abundant in rosemary (Rosmarinus officinalis) and lavender (*Lavandula stoechas*) (55). α-pinene is also the main compound found in frankincense *(Boswellia frereana)*, Mastic tree *(Pistacia lentiscus)*, myrtle *(Myrtus communis* and Leptospermum ericoides), rock rose *(Cistus creticus* and *salviifolius)*, juniper *(Juniperus communis)*, camphor *(Cinnamomum camphora)*, and conifers *(Pinaceae* family) (56). On the other hand, β-pinene is the main compound in balsam fir *(Abies balsamea)*, Guinea grains *(Aframomum angustifolium)*, and *Ferula gummosa* and *rubricaulis*, (56). Often used in perfume (57), pinene is also a major component of aromatherapy essential oils (58). Several plants high in pinene have been used in traditional medicines for a variety of conditions, such as gastrointestinal disturbances, seizures, inflammation, pain, snake bite, colds and fevers, hypertension, rheumatism, cancer, fungal infection, anxiety, and depression amongst others (59–62).

### Pinene Pharmacology

Pinene is a bicyclic monoterpene that consists of two isoprene units and has the molecular formula C_10_H_16_ (63). Differentiated by their physical structure, α-pinene is recognised for the alkene located inside the six-membered ring and β-pinene for its placement on the outside of the ring (Figure 5). Each structural isomer each has its own enantiomer, resulting in 4 pinene structures [(R)-(+)-α-pinene, (S)-(–)-α-pinene, (R)-(+)-β-pinene and (S)-(–)-β-pinene), and can be metabolised into a number of other terpenes, acids, aldehydes, and alcohols found in nature (55, 64).

The biological mechanisms of action of pinene remain unclear; however, the low molecular weight and high lipophilicity of monoterpenes, including pinene, suggest that it could penetrate the blood brain barrier. Indeed, α-pinene was detected in the mouse brain (extracts from whole brain homogenates) after 30 min of exposure via inhalation (50 μL/5 L air) (65). Interestingly, earlier work from the same authors showed that α-pinene levels were 2-fold higher when delivered as an oil blend (i.e., via inhalation in the presence of other essential oils: p-cymene, 1,8-cineole, and limonene) (66), suggesting that uptake of α-pinene by the brain can be influenced by the inclusion of other terpenes. One study examined the pharmacokinetics of (±) α-pinene isomers in 8 healthy males during 2 h of exposure by inhalation while engaging in low intensity exercise (50 W) (67). The results showed a 60% relative pulmonary uptake of α-pinene (i.e., concentration in inhaled vs. exhaled air), with blood levels that increased in a dose-dependent (450 mg/m^3^ > 225 mg/m^3^ > 10 mg/m^3^) linear manner. Blood levels rapidly declined following exposure termination (approximately 80% decrease within 30 min, and non-detectable levels after 4 h) (67), demonstrating that inhaled pinene is readily metabolised by the body.

In terms of safety, pinene isomers are approved for safe use in human food and cosmetic applications globally, and evidence shows that both α-pinene and β-pinene are non-toxic and non-mutagenic (68–70). However, several studies have reported upper airway irritation following pinene inhalation, including a clinical study that examined workplace exposure to high levels of pinene (71). They study found that irritation was reversed 6 h after exposure and further acute exposures did not cause deterioration suggesting no long-term detriments (71). On the other hand, oral dosing of α-pinene was effective in treating mucosal inflammation in a mouse model of allergic rhinitis through its ability to attenuate NF-kB signalling and mast cell activation (72).

α-pinene and β-pinene do not appear to have an appreciable binding affinity for CB1 or CB2 receptors (*in-vitro*) (10, 11). Pinene can exert effects on the brain through its ability to act as a positive modulator of the major inhibitory neurotransmitter, γ-aminobutyric acid (GABA)_A_ receptor subtype by binding to the benzodiazepine site to improve sleep in mice (73). Indeed, α-pinene (0.63 mM) potentiated the response of GABA_A_ receptors to GABA by ~49–57% compared to GABA alone, *in-vitro* (74). In addition, β-pinene targets the serotonin 5-HT_1A_, and β-adrenergic receptor sub-types to exert antidepressant effects in mice (75), suggesting that β-pinene may possess similar properties to existing anti-depressant drugs that target monoaminergic (eg serotonin and norepinephrine) signalling in the brain (76); however, further studies are needed to confirm. Furthermore, evidence suggests that pinene is neuroprotective, with anti-inflammatory and anti-oxidant properties, and may exert therapeutic benefits in the brain in multiple disease states.

### Pinene Anti-inflammatory, Antioxidant, and Neuroprotective Properties, *in-vitro* and *in-vivo*

Studies have shown that pinene protects against oxidative stress, inflammation, and neuronal damage, *in-vitro*. For example, in studies using mouse macrophages challenged with lipopolysaccharide (LPS), α-pinene reduced pro-inflammatory markers [interleukin-6 (IL-6), tumour necrosis factor-α (TNF-α), and nitric oxide (NO)] by suppressing mitogen-activated protein kinases (MAPKs) and the nuclear factor-kappa B (NF-κB) pathway (77, 78). These results demonstrate an ability of α-pinene to directly inhibit inflammatory signalling pathways in immune cells. Preclinical studies have also shown neuroprotective properties of α-pinene in models of multiple neurological illnesses *(in-vivo)*. For example, α-pinene (50 and 100 mg/kg) administered to mice immediately after the induction of ischemia [via middle cerebral artery occlusion followed by reperfusion (MCAOR)] decreased the size of the infarct and improved behavioural neurological scores that were attributed to anti-oxidant and anti-inflammatory effects in the hippocampus, cortex, and striatum (79). In addition, α-pinene metabolite, (S)-cis-verbenol, exhibited neuroprotective, anti-oxidant, and anti-inflammatory properties (reduced interleukin 1β and TNF-α in the cortex and striatum) in models of ischemia and stroke [including MCAOR (*in-vivo*) and neuronal oxygen-glucose deprivation (*in-vitro*)] (80). The same study revealed that (S)-cis-verbenol-induced calcium influx was not altered by N-Methyl- d-aspartic acid (NMDA) in cultured cortical neurons, suggesting no influence on glutamate receptor signalling, but protected against LPS-induced inflammation by suppressing pro-inflammatory cytokine (IL-1β, IL-6, TNF-α) mRNA expression in those cells (80). The anti-oxidant properties of α-pinene were also apparent in a rodent model of pentylenetetrazole (PTZ)-induced convulsions, as pre-treatment with α-pinene decreased seizure time and increased catalase and peroxidase enzyme activity; the latter of which prevent oxidative damage (81). Contrary to the findings of Zamyad et al. (81) and Felipe et al. (82) attributed the same therapeutic effects only to β-pinene in the PTZ model, with no benefit from α-pinene even though both α-pinene and β-pinene increased striatal dopamine concentrations (vs. untreated controls), which is interesting given the anti-convulsant properties of some compounds that stimulate striatal dopamine levels [see (83) for review]. Together, the evidence suggests that pinene can directly inhibit pro-inflammatory cell signalling pathways to suppress inflammatory response *in-vitro* and may confer anti-oxidant activity and neuroprotection in models of neurological illness, including ischemia, stroke, and seizures. However, the evidence is generally limited to single studies, justifying further robust experimentation.

### Pinene as an Analgesic in Inflammatory and Neuropathic Pain

α-pinene (5–25 mg/kg, orally) provided analgesia and reduced inflammation following exogenous application of irritants (carrageenan or complete Freund's adjuvant) that lasted for 48-h in mice (84). Pinene also reduced pain associated with migraine through its regulation of inflammation and vasoactive modulators (85). Repeated pinene treatment prevented mechanical sensitisation associated with partial ligation of the sciatic nerve in a rodent model, with efficacy similar to existing anti-inflammatory drugs used to treat neuropathic pain (indomethacin and gabapentin) (84). Compared to morphine, one study reported lower peripheral pain response in mice following α-pinene treatment, with longer lasting effects (86). The mechanisms underlying α-pinene-induced analgesia are unclear but may involve GABA_A_ and μ-opioid receptors, as Rahbar et al. (87) demonstrated that the ability of α-pinene (administered directly to the brain) to reduce capsaicin-induced nociception in dental pulp was prevented by pre-treatment with receptor antagonists, bicuculline or naloxone, respectively. However, the ability of pinene to reduce pain in inflammatory states and exert longer-lasting analgesia suggests that pinene may have a broader mechanism of action compared to existing opioids. Evidence is limited to single studies and more research is needed.

### Anti-depressant and Anxiolytic Effects of Pinene

Several rodent studies have examined the efficacy of α-pinene and β-pinene in treating anxiety- and depressive-like behaviours, with a consensus of positive findings. For example, β-pinene (100 mg/kg i.p.) exerted anti-depressant effects (increased mobility in the forced swim test) that were blocked by pre-treatment with antagonists of the serotonin 5-HT_1A_ receptor (WAY 100635), β adrenergic receptor (propranolol), and dopamine D_1_ receptor (SCH23390), and the noradrenergic neurotoxin (DSP-4) (75). These findings were echoed by Kasuya et al. (88), who reported anxiolytic behaviours in mice (increased time spent in the open arms of the elevated plus maze) following 60 and 90 min of exposure to a-pinene (10 μg/L air, via inhalation). In a similar study, the authors detected the presence of a-pinene in the brain (89) and reported significantly increased hippocampal brain-derived neurotrophic factor (BDNF) and tyrosine hydroxylase [the enzyme that catalyses the conversion of tyrosine to dopamine pre-cursor, Ldihydroxyphenylalanine (L-DOPA)] mRNA expression in the midbrain after 60-min of exposure (88). Another study reported a reduction in anxiety-like behaviour (in the elevated plus maze) and increased α-pinene concentrations in the brain and liver following exposure to α-pinene (10 μg/L air, via inhalation for 90-min/day for either 1, 3, or 5 days) compared to the controls (water vapour) (90). Interestingly, the α-pinene concentrations were significantly higher in the mouse brain prior to the elevated plus maze test compared to immediately after (in small sample size of *n* = 3–4/group) (90), suggesting that the anxiogenic test conditions may have increased metabolism of α-pinene in the brain; however, examination of α-pinene metabolism in larger sample sizes is required.

β-pinene (100 mg/kg, i.p.) increased mobility in the forced swim test significantly higher (~2-fold) than the traditional anti-depressant, imipramine (91). β-pinene did not induce detrimental effects on motor coordination (in the rotarod and traction tests) that were apparent in the diazepam treatment group, but caused a reduction in activity in the open field test compared to the controls (91). Two-weeks of treatment with α-pinene improved depressive-like behaviour (decreased immobility in the forced swim test) in Wistar-Kyoto rats (92), a rodent model of innate treat-resistant depression (93). The behavioural improvements were associated with increased markers of oxidative phosphorylation (mitochondrial function) in the hippocampus and pre-frontal cortex, and increased hippocampal parvalbumin mRNA expression in the α-pinene treatment group (92), suggesting that this terpene exerts effects on GABAergic signalling in the rat brain, which could contribute to its anti-depressant effects. Together, these findings suggest that the anti-depressant and anxiolytic effects of α-pinene and β-pinene may occur through mechanisms involving 5-HT_1A_, β-adrenergic and D_1_ receptors, as well as increased hippocampal BDNF and dopamine synthesis in the mid brain; systems that have been identified as key players in the pathogenesis of depression and anxiety (94, 95). However, confounding results exist as pinene decreased spontaneous activity in the open field test (91) demonstrating that further research is needed to elucidate the possible therapeutic and negative effects of these compounds.

### Effects of Pinene on Cognitive Impairment

In a mouse model of memory impairment induced by muscarinic receptor antagonist, scopolamine, a single acute dose of α-pinene (10 mg/kg, i.p.) significantly increased spontaneous alterations in the Y maze, improved spatial recognition in the Morris water maze and increased learning in the passive avoidance test (96). α-pinene significantly increased choline acetyltransferase (ChAT, which catalyses the production of ACh) mRNA expression in the cortex, but not acetylcholine esterase (AChE, which degrades ACh), M_1_ or M_2_ receptor levels, and increased hippocampal expression of anti-oxidant transcription factors (96). A lack of effect of α-pinene and β-pinene on AChE activity was also identified in an earlier report examining these enzymes in human erythrocytes (97). These results suggest that α-pinene may increase cortical acetylcholine production through ChAT activity and exert anti-oxidant effects in the hippocampus as a mechanism that contributes to its pro-cognitive effects. This response could be important when considering the cholinergic hypothesis of Alzheimer's disease, where amyloid beta plaques cause a significant loss of cholinergic neurons in the nucleus basalis of Meynert, diminished ChAT transcription and activity, and loss of cholinergic synapses (including in the hippocampus) that correlate with impaired attention and memory in Alzheimer's disease patients [reviewed in (98)]. In Drosophila melanogaster, administration of beta amyloid 42 (Aβ42) induced phenotypical alterations in the flies (i.e., Aβ42-induced rough eye phenotype) that were attenuated by α-pinene and β-pinene (as well as several other terpenes) (99). Together, the results provide limited evidence that pinene exerts effects on cholinergic neurotransmission and beta amyloid with relevance to Alzheimer's disease; however, rodent models of Alzheimer's disease are needed to examine neuropathological and cognitive behaviours. Furthermore, the pathophysiological relevance of altered beta amyloid protein must be elucidated as the role of plaques in Alzheimer's disease as either protective or detrimental is unclear (100).

### Pinene and Insomnia

Essential oils rich in α-pinene and β-pinene have reportedly been used in traditional medicine to aid sleep for centuries see (55), suggesting that these blends could support mental health due to the link between adequate sleep and reduced risk of mental ill health and improved symptoms (101). Yang et al. (73) examined the effects of α-pinene on the sleep-wake profiles of mice, reporting that non-rapid eye movement (REM) sleep duration was prolonged and sleep latency was reduced in a dose-dependent manner (i.e.,: efficacy was observed at 50 and 100 mg/kg i.p., but with less effect at lower doses 12.5 and 25 mg/kg). α-pinene also potentiated GABA_A_ receptor inhibition of post synaptic currents in the hippocampal CA1 region, which were blocked by an antagonist of the benzodiazepam binding site on the GABA_A_ receptor (flumazenil) (73). Pinene metabolites, myrtenol and verbenol have been identified as potent positive allosteric modulators of synaptic and extra-synaptic GABA_A_ receptors (102). Together, these results demonstrate that α-pinene can regulate GABAergic inhibitory tone by influencing GABA_A_ signalling, and may improve sleep by potentiating hippocampal GABAergic transmission through interactions with the GABA_A_ receptor benzodiazepine site. However, Yang et al. (73) reported that α-pinene did not alter REM sleep, contrary to another finding that pinene exposure by inhalation (60 ml of 0.3% w/w odorant solution) increased REM sleep in rats (103), with the apparent discrepancy attributed to differences in experimental methodologies (drug dose, delivery method, species). Clinical trials examining the efficacy and safety of isolated pinene isomers on sleep are also required.

### Effects of Pinene-Rich Plant-Derived Essential Oils: Anti-oxidant, Anti-inflammatory, Anxiolytic, Neuroprotective, and Pro-cognitive Properties

Studies show that a number of essential oils containing α-pinene as a major constituent exert therapeutic benefits relevant to brain health. For example, α-pinene-rich essential oil of Greek sage *(Salvia Fruticosa)* prevented oxidative stress and astrocyte cell death induced by hydrogen-peroxide; protective properties that were attributed to α-pinene and α-humulene (caryophyllene) (104), while β-pinene and β-pinene-rich essential oil from Spanish sage *(Salvia lavandulaefolia)* exhibited potent anti-oxidant effects against lipid peroxidation and anti-inflammatory activity in rat leukocytes (105). Furthermore, α-pinene-rich essential oil obtained from the leaves of *Ugni myricoides* [(Kunth) O. Berg) (5–25 mg/kg, orally], induced analgesic and anti-inflammatory effects following exogenous application of irritants (carrageenan or complete Freund's adjuvant) in mice (84). In another study, terpene-rich (e.g., α-pinene, β-pinene, limonene, linalool, and caryophyllene) essential oil from lemon *(Citrus limoni)* peel exhibited properties including metal chelating capacity, inhibition of lipid peroxidation, and AChE and butyrylcholinesterase (BChE) activities in rat brain homogenates (106); therapeutic properties with relevance to neurodegenerative disorders, such as Alzheimer's disease. Indeed, lemon essential oil administered to amyloid precursor protein/presenilin 1 (APP/PS1) transgenic mice (a model of Alzheimer's disease) also significantly improved performance in the novel object recognition and Morris water maze tests (suggestive of improved recognition and working memory) that were associated with reduced cortical amyloid protein precursor expression, downregulated hippocampal AChE activity and increased ACh levels, and increased expression of synaptic markers (synaptophysin and post-synaptic density protein 95 (PSD-95) in both the cortex and hippocampus of APP/PS1 mice compared to untreated controls (107). These positive effects of pinene-rich whole plant extracts on cholinergic neurotransmission, preservation of synaptic function, reduced amyloid beta deposition, and improved cognitive behaviours indicate relevance as a treatment for Alzheimer's disease worthy of further investigation.

Essential oil blends rich in pinene can also induce anxiolytic and anti-depressant effects in pre-clinical studies, for example, α-pinene-rich rosemary *(Rosmarinus Officinalis)* essential oil (via inhalation) induced anti-depressant and anxiolytic effects (i.e., increased mobility during the tail suspension test) that were associated with decreased serum corticosterone and elevated dopamine in the hippocampus and striatum (108). In addition, Moshgak *(Ducrosia anethifolia)* and shell ginger *(Alpinia zerumbet)* essential oils decreased anxiety-like behaviours (increased time spent in the open arms of the elevated plus maze) and increased brain α-pinene levels following inhalation in mice (109, 110). Another study reported a decrease in anxiety-like behaviours in the elevated plus maze, and the presence of α-pinene in the hippocampus, pre-frontal cortex, and hypothalamus of mice following inhalation of *Chamaecyparis obtusa* essential oil (90-min, 8 or 32 μL/L air) (89). *Litsea glauescens* essential oil (100 mg/kg, i.p.) used in Mexican Traditional Medicine to alleviate anxiety and depression (rich in β-pinene and linalool) increased mobility in the forced swim test ~2-fold greater than the traditional anti-depressant, imipramine (91). Although essential oil treatment was without motor coordination side-effects observed in the diazepam group, it reduced activity in the open field test compared to the controls (91), which could suggest negative effects on aspects of locomotor, exploratory and/or anxiety-like behaviours and requires further examination. Similarly, Roman chamomile (*Chamaemelum nobile)* rich in α-pinene (2-weeks exposure by inhalation) improved depressive-like behaviour (decreased immobility in the forced swim test) and increased mitochondrial function in the hippocampus and pre-frontal cortex, in a rodent model of innate treatment-resistant depression [Wistar-Kyoto rats (92, 93)]. Together, these findings demonstrate that α-pinene, as a component of essential oils, can penetrate the brain and induce alterations in behaviour, possibly via mechanisms involving regulation of dopamine and acetylcholine signalling, and anti-oxidant activity; however, this evidence is mostly limited to single studies. Further research into the mechanisms and clinical efficacy of these pinene-rich oils in people with anxiety are also required.

### Pinene-Conclusion and Considerations for Future Studies

A summary of evidence is depicted in Figure 5. Research shows that pinene can enter the brain following inhalation, oral and intraperitoneal administration methods. It exerts effects on multiple signalling pathways, including GABAergic, cholinergic, dopaminergic, serotoninergic, adrenergic, noradrenergic neurotransmitter systems, in multiple regions of the brain (e.g., hippocampus, frontal cortex, striatum, midbrain). Although limited, evidence suggests that pinene is anti-inflammatory and prevents oxidative stress, possesses anti-depressant, anxiolytic and anti-seizure properties, confers neuroprotection in models of stroke and ischemia, improves cognition, and provides analgesia in inflammatory, migraine-associated, and neuropathic pain in pre-clinical rodent or *in-vitro* settings. Several studies have shown that pinene has similar or better efficacy compared to existing medications for some indications. Overall, these studies require replication and the efficacy and safety of pinene for these indications remains unknown as examination of pinene in humans are lacking. Nevertheless, with indications of their psychopharmacological and anti-inflammatory effects, pinene isomers may be of use in psychiatric illnesses, while the combination of anti-depressant, anti-inflammatory, and nociceptive properties may make these compounds suitable as a treatment for chronic neurological conditions effecting the central nervous system, such as chronic pain-based conditions (e.g., fibromyalgia), but further research is needed.

## Linalool

**Figure 6:**
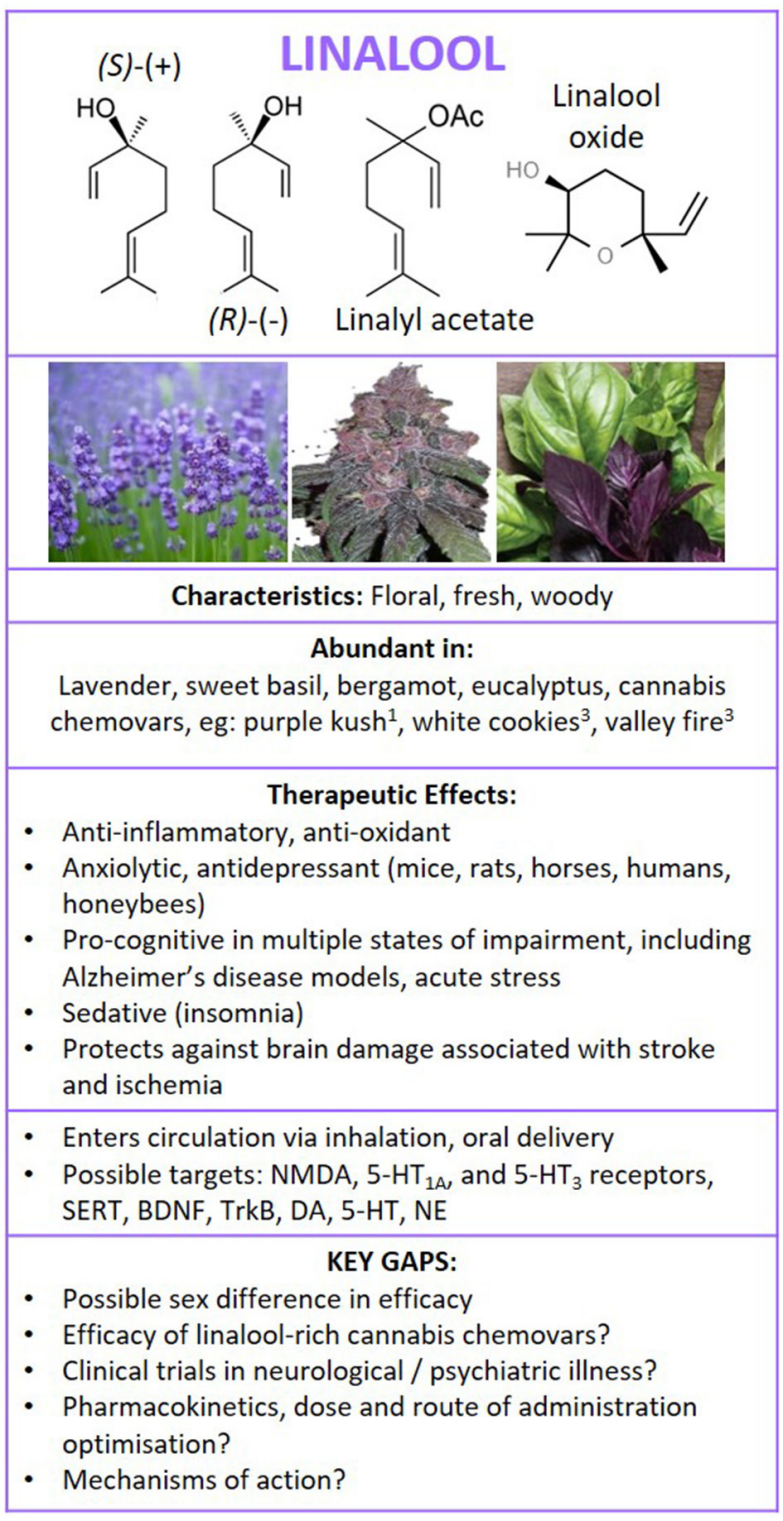
Structure of linalool isomers and derivatives. Summary of findings following literature search on the therapeutic effects of linalool in neurological and psychiatric illness, and brain health. ^1^Booth et al. (25) and ^3^Zager et al. (27). NMDA, glutamatergic N-methyl-D-aspartate receptor; 5HT1A, serotonin 5-HT1A receptor; 5HT3, serotonin 5-HT3 receptor; SERT, serotonin transporter; BDNF, brain-derived neurotrophic factor; TrkB, Tropomyosin receptor kinase B; DA, dopamine; 5-HT, serotonin; NE, norepinephrine.

Linalool is an acyclic monoterpene that exists as two enantiomers in nature, *(R)-*(–)-linalool and *(S)-*(+)-linalool (Figure 6). Linalool is a biosynthetic precursor to several other alcohols and aldehydes, therefore plants expressing linalool synthase enzyme also produce a number of linalool derivatives (111, 112), including linalyl acetate (also known as bergamol) and linalool oxide (Figure 6). Linalool is abundant in aromatic plants; however, differences in the ratio of enantiomers can yield different fragrances (113). For example, plants abundant in *(R)*-linalool, such as lavender, sweet basil, purple basil, bergamot and eucalyptus, produce a floral, fresh and woody scent, while *(S)*-linalool yields less robust fragrance similar to pettigrain, i.e., more subtle sweet, floral and woody tones (111, 113). Linalool is also abundant in cannabis chemovars, including Purple Kush (25) and S8.P38.BX.08 [detailed in Lewis et al. (52)].

### Linalool Pharmacology

Linalool, its derivatives and plant-derived oils rich in these compounds have been described as non-toxic in a number of studies (114–117), with no significant potential to produce genotoxic or mutagenic effects, and are non-irritating or sensitising to human skin when used as a fragrance (114). However, one study reported that linalool hydroxide (a linalool auto-oxidation product) increased oxidative stress *in-vitro*, which may have relevance to contact dermatitis (118). On the other hand, another study reported that linalool added to a collagen matrix accelerated wound healing, which was associated with decreased inflammation and scar formation in mouse skin tissue, *in-vivo* (119). A report by Cal (120) showed that linalool can be absorbed into the stratum corneum and epidermal layers of human skin, *ex-vivo*, with increased absorption between 1 and 4 h of exposure, and 10–20% loss from the skin per hour after exposure cessation. Following oral dosing (500 mg/kg) in rats, 1,2-[^14^C]linalool was absorbed from the gut and ~65 and 95% excreted within 24 and 48 h, respectively, via urinary, faecal and respiratory routes (121). Exposure to lavender oil in horses increased plasma linalool levels that reached maximum concentrations 20-min post-exposure, with low levels still measurable at the final testing time of 65 min (122). This finding was also noted in a case study involving a male subject who exhibited increased plasma linalool and linalyl acetate (100 and 121 ng/ml, respectively) following a 10 min abdominal massage with lavender oil, with levels that peaked at 19 min (123). In healthy humans (*n* = 7), inhalation of *(R)-*(–)-linalool resulted in higher plasma levels compared to dermal application, with maximum levels of 72.7 and 12.6 ng/ml, respectively, observed 40–45 min post exposure, and detectable levels ~25–30 min post-exposure through both routes of administration (124). Together, these results demonstrate that linalool administration (via oral, inhalation, or transdermal delivery) increases plasma levels of this terpene; however, the therapeutic relevance of these levels are unknown.

Linalool is a monoterpene with a low molecular weight and is highly lipophilic, suggesting that it may pass the blood brain barrier to exert effects on brain function; however, examination of brain levels of linalool following treatment are required. Linalool does not appear to have an appreciable binding affinity for typical endogenous cannabinoid receptor targets CB_1_, CB_2_, human transient receptor potential ankyrin 1 (hTRPA1) or human transient receptor potential vanilloid 1 (hTRPV1) receptors, *in-vitro* (10, 125). On the other hand, studies have indicated that linalool may target several other key neurotransmitter systems. For example, linalool was found to be a competitive antagonist of L-[^3^H]glutamate binding in rat cortical membranes in a dose dependent manner, with binding inhibited at 5 mM linalool (IC_50_ = 0.57) (126). In mice, linalool (350 mg/kg i.p.) delayed the onset of (without conferring protection against) seizures induced by NMDA (270 mg/kg NMDA s.c.), but protected against quinolinic acid-induced seizures (9.2 mM i.c.v.) in a dose-dependent manner [increasing protection (50–100%) with increasing dose (15–45 mM linalool i.c.v)] (126). Together those findings suggest an ability of linalool to block NMDA receptors, *in-vitro* and *in-vivo* (126). It is also a moderate antagonist of 5-HT-evoked currents (IC_50_ = 141 μM) in cells expressing the 5-HT_3_ receptor *(in-vitro)* and binds to the serotonin transporter (SERT, ~20% [^3^H]-citalopram binding apparent in the presence of 8 and 0.8 μL/ml linalool), but has no affinity for GABA_A_-benzodiazepine receptors (i.e., no detectable affinity during the [^3^H]-Ro 15-1788 binding assay) (115, 127). Overall, results show that linalool can exert effects on neurochemical signalling; however, further pharmaco-kinetic and -dynamic research is needed to understand the actions of linalool and metabolites on these signals in clinical neuropathologies.

### Neuroprotective, Anti-inflammatory, and Antioxidant Effects of Linalool

Evidence suggests that linalool exerts neuroprotective, anti-inflammatory, and antioxidant effects in the brain. For example, one study showed that linalool increased uncoupled mitochondrial respiration as a protective mechanism against glutamate hyper-stimulation in a neuronal cell line (HT-22) and in rodent hippocampal slices, resulting in reduced neuronal cell death (128). Linalool, linalyl acetate and lavender essential oil decreased TNF-α-induced inflammation in brain-derived endothelial cells (129) and markers of oxidative damage in neuronal (SH-SY5Y) cells exposed to hydrogen peroxide (115), demonstrating protection in the brain (*in-vitro*). Linalool also suppressed LPS-induced pro-inflammatory pathways and cytokine production (e.g., nitric oxide, NF-kB, TNF- α, IL-6, and IL-1β) in mouse macrophages (RAW 264.7 cells) and microglial (BV2) cells (130, 131), while other evidence showed that linalyl acetate was particularly effective at inhibiting pro-inflammatory histamine release from mast cells (*in-vitro*), compared to linalool and four other terpenes (132). These results demonstrate that linalool and its derivatives can directly suppress pro-inflammatory response from immune cells, *in-vitro*. Furthermore, pre-treatment with linalool protected against peripheral inflammation induced by exposure to the gramme negative bacterial endotoxin, *Salmonella typhimurium*, in mice (133). It also increased anti-inflammatory and antioxidant markers, and restored renal integrity in a streptozotocin-induced rat model of diabetic nephropathy (134). Overall, these studies demonstrate that linalool and its derivatives confer protection against inflammation and oxidative damage both in cell culture *(in-vitro)* and in rodent models *(in-vivo)*, suggesting that these compounds may be beneficial in treating inflammatory disease states with comorbid oxidative stress in patients; however, further research is needed to confirm.

### Linalool: Effects on Cognition in Models of Alzheimer's Disease, Stroke, and Ischemia

In an aged (21–24 months) triple transgenic mouse model of Alzheimer's disease (3xTg-AD), linalool (25 mg/kg, administered daily for 3 months) improved learning and memory in the Morris water maze and anxiety-like behaviours in the elevated plus maze, and reduced the amyloid load, tauopathy, and neuroinflammation in the hippocampus and amygdala (135). Similar results were reported in mice administered amyloid beta 1-40 (Aβ1-40), where linalool (100 mg/kg, i.p.) increased cognitive behaviours in the Morris water maze and reversed hippocampal levels of oxidative stress markers and apoptosis (136). In addition, lavender oil and linalool (100 mg/kg) prevented learning and spatial memory deficits, and oxidative stress induced by chronic D–galactose and aluminium trichloride administration (137). A number of alterations in the brain, including decreased acetylcholinesterase activity, increased hippocampal BDNF and Tropomyosin receptor kinase B (TrkB) expression, accompanied the behavioural improvements (137), suggesting that linalool may improve cholinergic signalling and synaptic plasticity in this model of cognitive impairment resembling Alzheimer's disease.

In a rat model of ischemia (MCAOR), intranasal administration of linalool (25 mg/kg) reduced infarct volume measured 1- and 7-days post stroke, with improved neurological scores and spatial memory (in the Morris water maze) compared to controls (138). Linalool-treated rats exhibited reduced cortical and hippocampal inflammatory markers and microglial activity. Interestingly, examination of bioavailability showed that intranasal delivery resulted in more rapid appearance of linalool in the plasma than intraperitoneal injection in these rats (138). In another study, oral linalool treatment (25 mg/kg/day, 1 month) improved neurological scores, motor function (in the Rotarod test), and cognitive function (via Morris water maze) in the MCAOR rat model of ischemia through mechanisms involving anti-inflammatory and anti-oxidant effects, and restoration of lipid homeostasis in the hippocampus (139). A similar result was noted by Park et al. (140), where linalool was protective against oxidative stress and inflammation following oxygen-glucose deprivation/reoxygenation-induced cortical neuronal injury (an *in-vitro* model of ischemic stroke). Overall, several studies have shown beneficial effects of linalool on brain function relevant to the treatment of Alzheimer's disease, ischemia and stroke; however, clinical trials are required.

### Effects of Linalool on Anxiety and Depression

Evidence suggests that linalool may be beneficial for social anxiety, as acute administration of linalool (100 mg/kg, i.p.) decreased anxiety-like behaviours in mice in the elevated plus maze, and restored social interaction in mice subjected to a social defeat paradigm, compared to socially stressed mice administered saline (controls) (141). In REM-sleep deprived mice, linalool improved hippocampal-dependent learning and memory behaviours (examined using the Y maze and passive avoidance tests), and exerted anti-depressant effects (i.e., decreased immobility in the forced swim test) (142). These improvements in behavioural outcomes were accompanied by a reduction in plasma cortisol levels in the linalool-treated sleep deprived mice compared to the controls (142).

Inhalation of linalool oxide (0.65, 1.25, 2.5, and 5.0%, for 7 min, *n* = 8/group) exerted anxiolytic effects in mice examined in the elevated plus maze (vs. vehicle-treated controls), particularly in the higher dose (5.0%) group, which showed similar efficacy to the benzodiazepam-treated (0.5 mg/kg, i.p.) positive controls (143). The study also reported a transient reduction in motor function (in the rotarod test) in rodents administered benzodiazepam after 30 min that was not apparent in the linalool oxide-treated groups (143). The efficacy of inhaled linalool (1 and 3% linalool, 60 min exposure) was also reported by Linck et al. (144), who observed reduced anxiety in the light/dark box test and aggressive behaviours, and increased social interaction in linalool-treated mice, with results comparable to the diazepam treatment group. These results present interesting implications for the use of linalool as an alternative to benzodiazepam for the treatment of various behaviours associated with anxiety.

In young mice (4-week old), linalool (500 mg/kg) increased exploratory, and decreased anxiety-like behaviours in the open field and elevated plus maze tests after 14-days of treatment, without affecting overall motor activity (145). These behavioural changes were associated with decreased 5-HT levels and a corresponding increase in the levels of serotonin metabolite 5-hydroxyindoleacetic acid (5-HIAA) in the pre-frontal cortex, hippocampus and striatum (145), demonstrating potential metabolism of 5-HT by linalool. There was also an increase in striatal dopamine [with no change in dopamine metabolite, 3,4-dihydroxyphenylacetic acid (DOPAC)] and reduced norepinephrine in the treated mice compared to the anxiogenic controls (145). Guzmán-Gutiérrez et al. (75) showed antidepressant-like effects (i.e., increased mobility in the forced swim test) following linalool administration that were blocked by pre-treatment with 5-HT_1A_ receptor antagonist, WAY 100635, but not 5-HT synthesis inhibitor, para-chlorophenylalanine, demonstrating a role for the 5-HT1A receptor in the antidepressant mechanisms of action of linalool. Another study reported reduced immobility of mice in the tail suspension test following (–)-linalool in a dose-dependent manner (100 and 200 mg/kg i.p., not 10, 50 mg/kg i.p.) (146). Mice exposed to linalool [20, 200, or 2,000 μL via inhalation in an enclosed box for 30 min compared to controls (*n* = 10/group)] also exhibited a dose-dependent decrease in anxiety-like behaviours in the light/dark box test and elevated plus maze, with efficacy that was blocked by GABA_A_ receptor antagonist, flumazenil (147). These results demonstrate that linalool induces antidepressant and anti-anxiety –like behaviours and influence monoaminergic neurotransmitter systems in key regions of the brain implicated in anxiety, depression and cognitive function in pre-clinical studies *(in-vivo)*.

In honey bees, linalool attenuated the aggressive response of soldier bees to the attack pheromones, which are produced by guard bees patrolling the hive to alert the solider bees of an imminent threat. Linalool reduced the pheromone-induced aggressive behaviours in the honeybee through a mechanism that diverted attention toward appetite behaviours in response to the floral scent (148). In horses, lavender oil inhalation (i.e., oil placed on nostrils) reduced indicators of stress-related behaviours, i.e.,: decreased heart rate and number of defecations in the presence of a novel object, alert postures in response to a social isolation event, and heart rate in response to mild fright (sudden appearance of an unfamiliar object in the stall), with measurable plasma linalool levels following treatment (122). Therefore, the anxiolytic effects of linalool have been reported across a range of species; however, replication is required.

### Effects of Linalool-Rich Essential Oils: Effects on Inflammation, Anxiety, Depression, and Cognition

Studies show that essential oil from linalool-rich *Citrus aurantium* peel and flower (neroli) extracts inhibit LPS-induced inflammatory response in macrophages by supressing biomarkers, including TNF-α, NF-kB, nitric oxide and interleukins−6 and−1β *(in-vitro)* (149, 150). Neroli extract also reduced pain-associated behaviours and inflammation in mice and rats (151). A similar analgesic and anti-inflammatory effect was observed in rats administered linalool-rich *Zhumeria majdae* essential oil, native to Iran (152).

Linalool-rich plant-derived oils also exert antidepressant, anti-anxiety and pro-cognitive effects in rodent models. For example, male mice administered linalool-rich *Cananga odorata* (ylang-ylang) essential oil exhibited significant reductions in anxiety-like behaviours in the elevated plus maze and light-dark box tests, and increased hippocampal 5-HT concentrations; however, female mice showed less treatment response (153), suggesting a sex-dependant effect that requires further investigation. *Litsea glaucescens* (Lauraceae) used in Mexican Traditional Medicine to relieve “epilepsy, fright and sadness” induced anti-depressant effects in the forced swim test in mice that were attributed to high levels of linalool and β-pinene (91). In addition, essential oil of bergamot (containing 38% d-limonene and 30% linalyl acetate as major components) administered to healthy rats induced a dose-dependent effect on electroencephalogram (EEG) and locomotor activity, i.e., low doses reduced locomotion and increased hippocampal and cortical delta wave amplitude, while higher frequencies, lower amplitudes, and greater mobility were observed in with higher doses (154). This result suggests an effect on the ascending arousal pathways in the brain, involving multiple neurotransmitter signalling systems (noradrenergic, serotonergic, cholinergic and histaminergic) (154, 155). In another study, *Lavendula angustifolia* essential oil (200 mg/kg, i.p.) decreased anxiety-like behaviours a mouse paradigm of social defeat, suggesting benefits for social anxiety (141). In addition, linalool-rich *Cinnamomum osmophloeum* oil (500 mg/kg for 14 days) increased exploratory, and decreased anxiety-like behaviours in the open field and elevated plus maze tests in young (4-week old) mice (145). The treatment increased striatal dopamine levels and 5-HIAA levels in the pre-frontal cortex, hippocampus, and striatum (indicative of serotonin metabolism in these regions), and decreased norepinephrine levels compared to the anxiogenic controls (145). On the contrary, another study reported that while anti-anxiety effects were induced by acute linalool (400–600 mg/kg, i.p. single dose administered 20 min prior to testing), there was no efficacy observed in rodents administered lavender oil (156). The reasons behind the lack of anxiolytic efficacy in lavender oil in that study are unclear; however, the study did not report the level of linalool present in the lavender oil (156). This is important as Takahashi et al. (157) reported significant differences in the terpene profile of 6 different species of lavender, including levels of linalool and linalyl acetate. Takahashi et al. (157) also reported moderate positive correlations between time spent in the open arm of the elevated plus maze, and linalool or linalyl acetate plant concentrations (*r* = 0.54 and 0.72, respectively). Therefore, plant strain and subsequent alterations to the ratio of linalool and linalyl acetate could be a consideration when investigating the therapeutic effects of plant-based extracts.

Several clinical studies have investigated the effects of linalool-rich oils in human subjects. The safety and efficacy of lavender oil capsules (Silexan™), which contain 36.8% linalool and 34.2% linalyl acetate, were examined in a recent network meta-analysis of clinical studies containing 645 subjects across five studies (158). The analysis revealed that the lavender oil reduced Hamilton Anxiety Scale (HAMA) total scores, with effects greater than, or equal to the traditional antidepressant drug, paroxetine, at doses of 160 and 80 mg, respectively (158). The study noted that there were no serious adverse effects; however, gastrointestinal dysfunction (nausea, diarrhoea, breath odour, and eructation) was reported in a small portion (1.2–10%) of patients. Furthermore, while fatigue was apparent in up to 16.2% of patients administered the traditional anti-anxiety medication, lorazepam, in one study, there were no reports of fatigue across studies following the lavender oil treatment (158). Another clinical trial examined the effect of petitgrain oil (containing linalyl acetate, linalool, and myrcene) on various neurophysiological parameters in adults (*n* = 42) subjected to an online task (159). Outcomes were measured pre- and post- task, with no significant differences in parameters between the treatment group and the controls during the pre-test phase. However, increased task performance speed, decreased anxiety, and improved mood (using Stait-Trait Anxiety Inventory [STAI] and the Profile of Mood States [POMS]) were noted in the group that performed the task in the presence of the petitgrain oil fragrance compared to the neutral oil (almond oil) controls (159).

### Linalool-Conclusion and Consideration for Future Studies

There is a body of evidence (mostly preclinical and *in-vitro* studies) demonstrating that linalool exerts neuroprotection against various stressors, can directly inhibit pro-inflammatory pathways and exert antioxidant effects in disease states, such as models of Alzheimer's disease, diabetic nephropathy, systemic inflammation following bacterial infection, stroke, ischemia, and inflammatory pain (Figure 6). In models of Alzheimer's disease, linalool improved cognitive behaviours and decreased oxidative stress, inflammation and amyloid load. In models of stroke and ischemia, linalool decreased necrosis, and improved neurological scores and cognitive function. Linalool and linalool-rich essential oils exerted anxiolytic, anti-depressant, and pro-cognitive benefits in human clinical trials (albeit limited in number) and other species, including mice, rats, honeybees, and horses. Linalool also conferred cognitive, anti-anxiety and anti-depressant benefits in preclinical modelling of sleep deprivation. Evidence suggested that the efficacy of linalool equals existing commercial anti-anxiety, analgesic, and anti-depressant medications (including lorazepam, benzodiazepam, and paroxetine) in some instances, often with lower adverse effects profile; however, well-designed clinical trials are required to confirm. The mechanisms underpinning the benefits of linalool also require further investigation; however, linalool appears to be an antagonist of NMDA and 5-HT_3_ receptors, with low affinity for GABA_A_, CB_1_, CB_2_ and TRPV receptors, it deceases AChE expression, and increases BDNF and its TrkB receptor. Studies also show that linalool exerts effects in the hippocampus, striatum and prefrontal cortex. Beneficial effects of linalool were apparent following dosing by oral, i.p., and inhalation and trans-dermal routes of administration; however, some evidence suggested that the response is sensitive to dose. Furthermore, the ratio of linalool to its derivatives across different plant species (i.e., different species of lavender plants) could influence efficacy. Further investigation into the efficacy, pharmacokinetics, and long-term safety of linalool in humans is required, with consideration of potential of sex differences. In addition, studies examining linalool-dominant cannabis chemovars are needed. This is particularly important given that other cannabis molecules, particularly CBD and THC confer benefits for a number of illnesses described in this section. Therefore, the approach of a linalool-rich whole plant cannabis extract could confer further benefits through synergism (i.e., multi-targeted approach for enhanced therapeutic effect).

## Overall Conclusion and Future Directions

Terpenes have been used in Traditional medicines for centuries; however, recent botanical and medical research is beginning to provide evidence for the potential use of plant-derived terpenes in modern medicine. Based on the evidence presented in this review, such an approach has potential to lead to the discovery of a plethora of novel therapeutics for chronic illnesses, including neurological and neuropsychiatric disorders for which existing medications can be inadequate in alleviating symptoms. Indeed, evidence supporting the health benefits of forest volatile organic compounds rich in terpenes such as pinene, linalool, limonene and caryophyllene, could provide an alternative approach to positive outcomes for well-being and mental health, with implications for public health and even community landscape design.

This review has identified a line of evidence in the scientific literature that supports the positioning of specific plant-derived terpenes, pinene, and linalool (both in isolation and as major components of botanical extracts) as key candidates for further research as novel medicines for an array of brain illnesses. This includes therapeutic potential in stroke and cerebral ischemia, inflammatory and neuropathic pain (including migraine), cognitive impairment (including models of Alzheimer's disease and ageing-related cognition), insomnia (and associated cognitive impairment and anxiety), anxiety (including social anxiety), and depression. There is some evidence that these terpenes provide therapeutic efficacy similar to existing commercial medications for several indications, including analgesics, anti-inflammatories, anti-anxiety and anti-depressant drugs, with fewer adverse effects e.g., sedation and motor impairment. The mechanisms by which pinene and linalool exert their effects are largely unknown; however, evidence shows that these molecules enter the circulation and/or brain (via multiple routes of administration: inhalation, oral, i.p.) and alter neurochemical and neurotrophic signalling in discrete regions of the brain implicated in cognition, anxiety and depression, including the hippocampus, frontal cortex, striatum, and midbrain. However, this should be interpreted with caution as a therapeutic concentration of pinene or linalool in the brain or circulation for any indication is unknown. Pinene and linalool influence GABA, glutamate, serotonin, dopamine, acetylcholine, as well as inflammatory, oxidative stress, and neurotrophic (BNDF) pathways in the brain. Although limited, the evidence showing that these terpenes alter brain function and change cognitive and affective behaviours demonstrates that pinene and linalool are, by definition, psychoactive compounds.

Other important questions require further investigation, such as the efficacy of terpenes (including cannabis-derived) in humans. Other than a handful of studies in healthy individuals, clinical trials are mostly lacking, particularly in patients with psychiatric/neurological illnesses. Future investigations should include pharmacokinetic studies to inform optimal dosage and route of administration in specific populations, as well as examination of the long-term safety and efficacy as chronic treatments for these often life-long illnesses. For example, biogenic volatile organic compounds derived from plant and essential oils can induce detrimental respiratory and dermatological effects, such as asthma, allergic rhinitis and eczema, in sensitive patients (160). These effects should be considered in efforts to balance the benefits and potential harms of terpenes for medicinal use. Studies may potentially extend to examination of terpene efficacy in animal health [e.g., linalool exerts anxiolytic effects in horses and honeybees (122, 148)]. Future research should emphasise examining treatment effects in both sexes, as most existing studies are male-dominant, while evidence demonstrates that medication therapeutic response, side-effects profiles and disease pathophysiology are effected by sex (93, 161–163).

A number of cannabis chemovars express high levels of pinene and linalool; however, to-date, the efficacy of cannabis-derived terpenes for brain health, either as isolated compounds, or as a whole plant extract, has not been examined in neuropathological states. The latter could yield interesting findings as a combination of beneficial compounds in cannabis (e.g., terpenes and CBD) could improve the efficacy above levels observed using the isolated compound through synergistic interactions in the brain. Furthermore, current understanding of the terpene capacity in cannabis plants is still developing, with 13 new terpene synthase enzymes recently characterised across 5 cannabis chemovars (25). Analytical studies show that cannabis chemovars not only possess distinct cannabinoid characteristics, but can be classified based on distinct terpene profiles (25–27, 164). As with every living organism, the genetics of a plant dictates its potential characteristics; however characteristics are also influenced by environmental factors (e.g., growing conditions, e.g., nutrition, humidity, light, as well as stressors such as predation), with differences across plant organs and time of harvest (25, 164, 165). Such conditions may affect translation of genetic code into functional proteins, e.g.: enzymes that support the reactions favouring pathways that produce specific terpene profiles. Therefore, it may be possible to optimise cannabis chemovars for specific illnesses through environmental manipulation, providing a unique opportunity to develop personalised medicines to treat disorders of the brain through a bidirectional translational interface between medical and horticultural sciences. Overall, it appears that the importance of the terpene profile of plants to humans extends further than mere olfactory and gustatory delight. Rather, these compounds have the potential for use as treatments for serious chronic neurological and psychiatric illnesses.

## Author Contributions

KW-G, HC, and CJN conducted reviews of the literature and drafted the manuscript. KW-G finalised the manuscript with inputs from HC and CJN. All authors contributed to the article and approved the submitted version.

## Conflict of Interest

The authors declare that the research was conducted in the absence of any commercial or financial relationships that could be construed as a potential conflict of interest.

## Publisher's Note

All claims expressed in this article are solely those of the authors and do not necessarily represent those of their affiliated organizations, or those of the publisher, the editors and the reviewers. Any product that may be evaluated in this article, or claim that may be made by its manufacturer, is not guaranteed or endorsed by the publisher.
